# Electrochemical cell recharging by solvent separation and transfer processes

**DOI:** 10.1038/s41598-022-07573-x

**Published:** 2022-03-08

**Authors:** Yohei Matsui, Makoto Kawase, Takahiro Suzuki, Shohji Tsushima

**Affiliations:** 1 Energy Chemistry Division, Energy Transformation Research Laboratory, Central Research Institute of Electric Power Industry, Yokosuka, 240-0196 Japan; 2 Department of Mechanical Engineering, Graduate School of Engineering, Osaka University, Suita, 565-0871 Japan

**Keywords:** Electrochemistry, Energy, Energy science and technology

## Abstract

Electrochemical conversion and storage of unutilized renewable energy will contribute to decarbonization. Here, we create the concept of a liquid electrochemical cell that discharges between the anodic and cathodic sides by reverse reactions of the same redox couple in different solvation states, which are created by differences in the mixture ratios of two solvents called the main solvent (MS) and the transferred solvent (TS). The cell can be charged by a transfer of the TS between the discharged anolyte and catholyte. As an example, we demonstrate a cell utilizing a ferro-/ferricyanide redox couple. Stable discharging and charging via the proposed method is achieved by utilizing water (MS) and acetone (TS). Additionally, dominating factors in the design of a high-performance system are discussed, focusing on the electron acceptability of the MS and the TS. The cell voltages are successfully tuned, and a cell voltage of 0.63 V is achieved by the combination of dimethyl sulfoxide (MS) and water (TS). Moreover, the cell can be customized by various electrochemical reaction systems, which can allow multiple options for the charging processes. This concept provides new approaches for the utilization of diverse energy sources as an input for the charging of electrochemical cells.

## Introduction

The utilization of diverse carbon-free energy sources is a crucial challenge for the decarbonization of energy systems. Electrochemical conversion and storage systems have received much attention for the efficient use of energy sources. For instance, electrochemical batteries are now in widespread use for the storage of electrical energy generated by various forms of renewable energy. The application of redox flow batteries or sodium-sulphur batteries is a promising method for large-scale energy storage^1–3^. Lithium-ion batteries and nickel-hydrogen batteries have been used in vehicles and mobile devices^4–6^. However, electrical energy must be provided to charge these conventional batteries since charging is generally achieved by reversing the redox reactions. Electrochemical cells that can be regenerated via different routes from conventional batteries may enable the direct use of other forms of energy as an input for charging. For the utilization of low-grade thermal energy sources, such as geothermal energy, waste heat or solar heat^7–9^, studies on thermoelectrochemical conversion systems have been conducted, such as thermoelectrochemical cells^10–15^, thermally regenerative electrochemical cycles^16,17^, and reverse electrodialysis systems^18,19^. On the other hand, there are also many kinds of renewable mechanical energy sources, such as wind energy, wave energy, tidal energy and small-scale hydroenergy^20–23^. To broaden the field of applications of electrochemical devices in energy systems, electrochemical cells with totally new concepts should be pursued.

**Figure 1:**
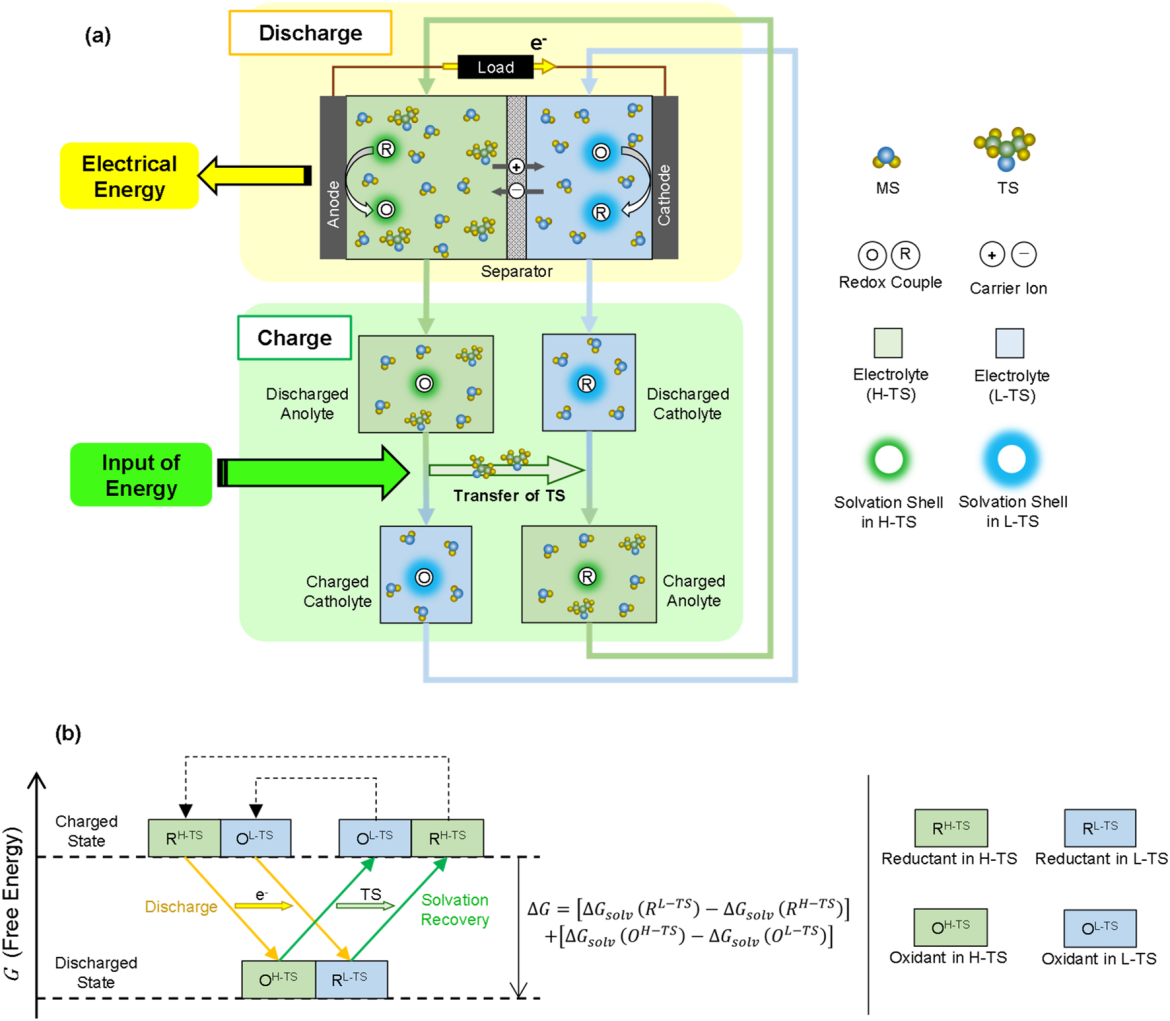
Concept of SDCs. (**a**) Schematic diagram of discharge and charge (solvation recovery) of SDC. (**b**) Change in state and free energy caused by discharge and solvation recovery. Δ*G*_*solv*_ (*R*^*H-TS*^) represents the solvation free energy in H-TS for the reductant. The solvation free energy in L-TS or for the oxidant is also represented in the same way.

Here, we create a novel concept for a liquid electrochemical cell called the solvation difference cell (SDC). In an SDC, the electrolyte solvent is composed of a mixture of at least two components called the main solvent (MS) and the transferred solvent (TS), but there is a difference in the mixture ratio between the anodic and cathodic sides (Fig. 1a). One electrolyte utilizes a solvent with a higher TS ratio (H-TS), whereas the other utilizes a solvent with a lower TS ratio (L-TS). Here, as an example, the anodic solvent is described as H-TS, and the cathodic solvent is described as L-TS. Anodic and cathodic reactions are reverse redox reactions of the same redox couple (R and O). Although this system appears to be similar to general concentration cells^24–27^, the installation of the TS leads to differences in the solvation states of R and O between H-TS and L-TS and contributes to a larger cell voltage. Note that the directions of the reactions are dependent on the components applied as a redox couple, MS, TS or other solutes.

Another remarkable characteristic of a SDC is its unique charging process called solvation recovery. The change in state caused by discharge can be recovered via different routes from the discharging process (Fig. 1a). Because the difference between the anodic and cathodic solvents is the amount of TS, the transfer of TS can result in a switch between the two solvents. After solvation recovery, the discharged anolyte can be reused as a fresh catholyte, and vice versa. Energy input is required for the transfer of the TS because the transfer between the discharged electrolytes does not proceed simultaneously.

Although the routes for discharging and solvation recovery are different, both routes are thermodynamically equivalent to the transfer of R and O between H-TS and L-TS (Fig. 1b). The transfer of redox species from one solvent to the other results in a change in free energy due to the difference in solvation free energy for the redox species between the two solvents^28–33^. Hence, the change in free energy due to the discharge and solvation recovery can be described using the difference in solvation free energy between H-TS and L-TS for R and O (Fig. 1b). Therefore, discharging a SDC can be generally considered as a conversion of the difference in the solvation free energy between the anodic and cathodic solvents for redox couples into electrical energy, and solvation recovery can be considered recovery of the change in the free energy by the transfer of the TS. Furthermore, considering that the method used for the separation and transfer of the TS is optional thermodynamically, diverse forms of energy can be used as an input for charging in accordance with the selected processes. In this work, we show an example of SDCs and discharging and charging via the solvation recovery process to show the viability of the concept. Moreover, dominating factors to design an SDC with a large cell voltage are discussed based on a solution chemistry-based strategy.

## Results

### Demonstration of the concept of a SDC

**Figure 2:**
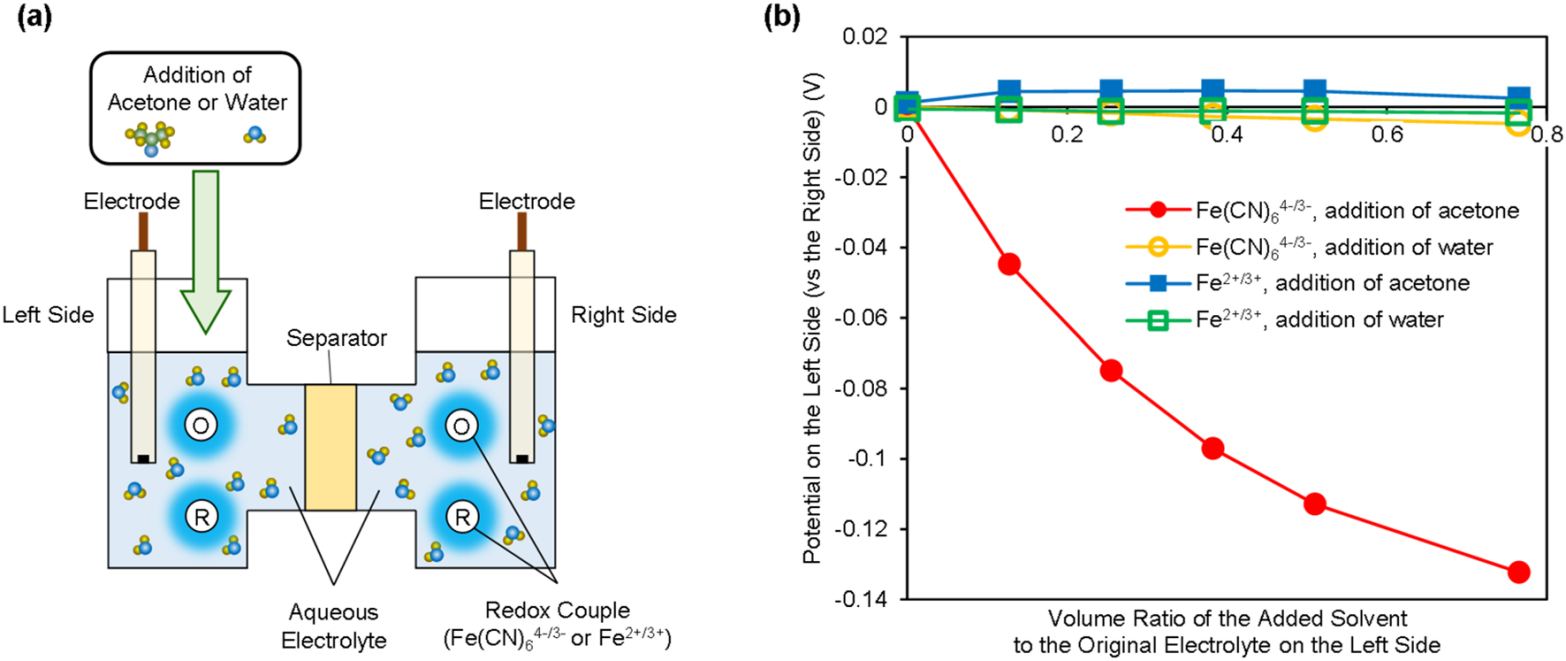
Demonstration of cell voltages generated by the addition of the TS (acetone). (**a**) Experimental setup used for the demonstration. (**b**) Change in the redox potentials of the ferro-/ferricyanide ions or ferrous/ferric ions due to the addition of solvents (acetone or water). The oxidant and reductant concentrations were both 50 mM, whereas those on the left side were lower due to the addition of solvents.

We experimentally show open-circuit voltages (OCVs) for SDCs generated by the addition of the TS with a H-shaped cell (Figs. 2a and S1). Under the initial conditions, the same aqueous electrolytes are prepared and set in both rooms of the H-shaped cell, which are separated by a glass filter. The ferrocyanide/ferricyanide redox couple (Fe(CN)_6_^4−/3−^, ammonium) or ferrous/ferric redox couple (Fe^2+/3+^, chloride) salts are dissolved in the electrolytes. We investigated the changes in the redox potentials observed following the addition of solvent (acetone or water) only to the left side of the cell shown in Fig. 2a. For the cells with the ferro-/ferricyanide redox couple, a large negative shift of the redox potential is observed due to the addition of acetone to the left side of the cell, whereas obvious changes are not observed when water is added (Fig. 2b). Because the maximum volume ratio of the solvent added to the original electrolyte is 0.77, the addition of water results in only a small dilution of the electrolyte. On the other hand, the addition of acetone leads to a difference in the solvent components on the right side (L-TS side) and left side (H-TS side), implying that the addition of acetone (TS) results in a change in the solvation states of the redox couple on the H-TS side and contributes to a change in the redox potential. A maximum shift for the redox potential of − 0.13 V is observed when the maximum volume ratio (0.77) of acetone is added, where the molar ratio of acetone to the total solvent on the H-TS side is 0.16. Additionally, similar changes in the redox potentials are also observed in cells with different concentrations of the ferro-/ferricyanide redox couple (Fig. S2). The differences in the redox potentials between the H-TS side and L-TS side can be considered the OCVs of the SDCs. For the cells with the ferrous/ferric redox couple, however, obvious changes in the redox potentials are not observed following the addition of acetone or water (Fig. 2b). These results imply that the OCVs of SDCs largely depend on what redox couple is applied because the effects of solvent components on the solvation free energy generally vary between redox species^28–32^.

**Figure 3:**
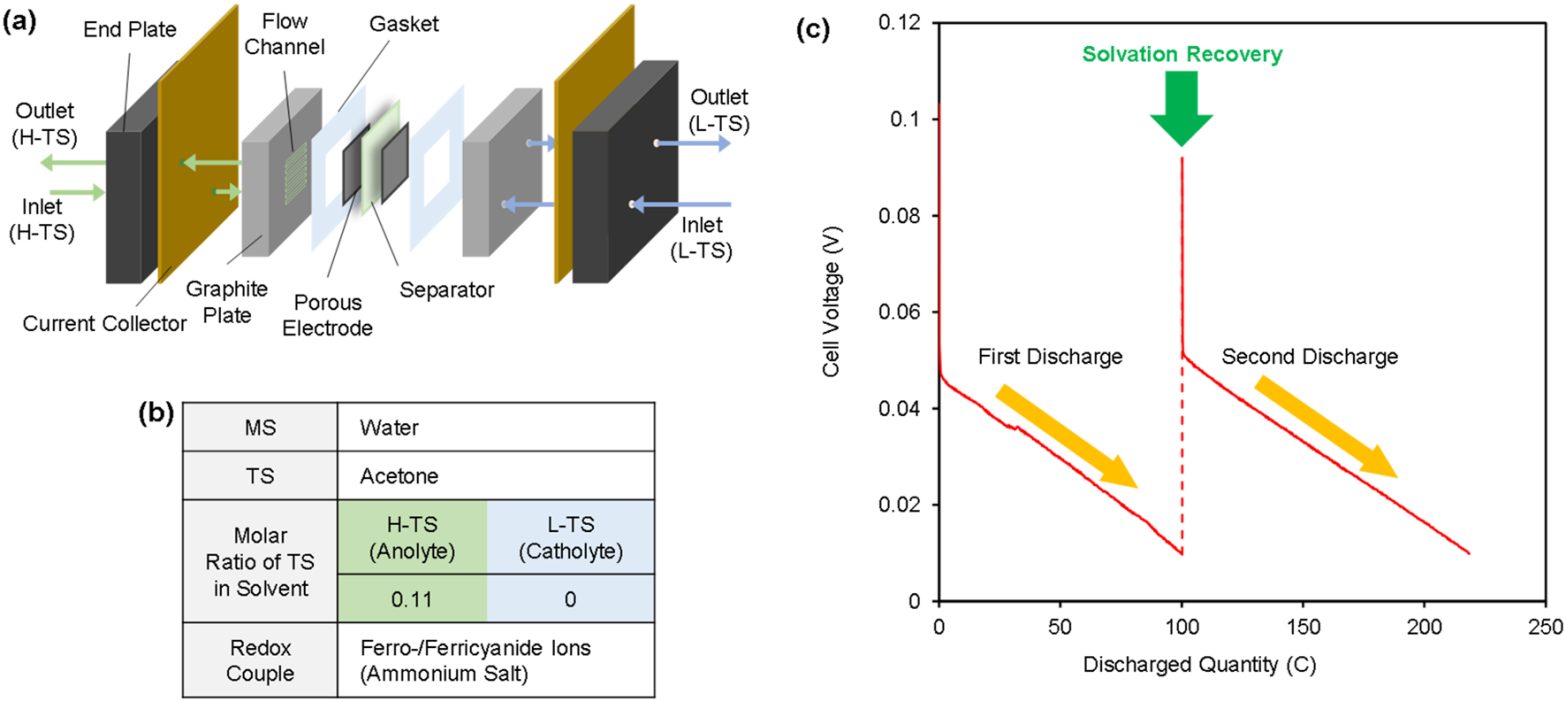
Demonstration of discharging and charging via the solvation recovery by the phase change process of the TS. (**a**) Structure of the flow-type cell used for discharging. (**b**) Components of the electrolytes. (**c**) The first and second discharging behaviours after the solvation recovery at a constant current density of 80 A m^-2^. The concentration of ferro-/ferricyanide salts in the catholyte is 200 mM, respectively, whereas that in the anolyte is lower due to the addition of the TS.

We also demonstrated discharging and charging via the solvation recovery process. The discharging test was conducted with a flow-type cell (Figs. 3a and S3). Carbon papers were applied to both the anodic and cathodic electrodes. The electrodes were separated by a cation exchange membrane. The anolyte and catholyte, which are circulated by peristaltic pumps, pass through serpentine channels in the cell during the discharging test. Moreover, the discharged electrolytes are regenerated by solvation recovery and reused in the second discharge. The solvation recovery was conducted by evaporation and condensation of the TS using a simple aqueous system (Fig. S4). The components of the electrolytes applied to the cell are listed (Fig. 3b). Ammonium salts of ferro-/ferricyanide ions were used due to their high solubility in the MS (water)^34^. Acetone was used as the TS in this cell because the boiling point of acetone is 56 °C, which is much lower than that of the water used as the MS. A large difference in the boiling point between the TS and the MS is preferred for solvation recovery by the phase change process that can be performed by low-grade thermal energy. In this cell, the H-TS side works as an anodic side because the addition of acetone leads to a negative shift in the redox potential, as shown in Fig. 2b. The cell shows stable discharging processes in which the cell voltages are decreased gradually as the ferro-/ferricyanide ions are consumed on the anodic and cathodic sides (Fig. 3c). Moreover, the electrolytes after the solvation recovery also achieve stable discharging equal to the initial value. This result indicates that the discharged SDC is successfully charged by the transfer of the TS from the discharged anolyte to the discharged catholyte. These results provide assurance for the concept of solvation recovery.

### Design of electrochemical reaction systems for large cell voltages

**Figure 4:**
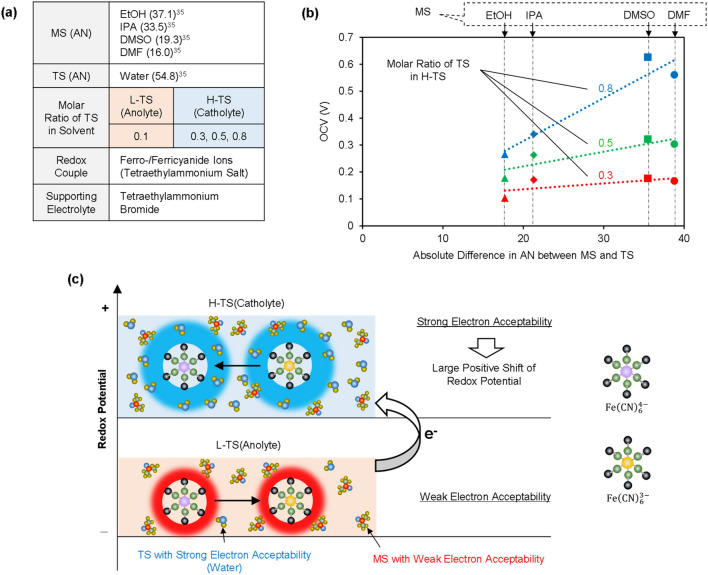
Example of designs for the electrochemical reaction system for large cell voltages. (**a**) Components of the electrolytes used for the measurement of the OCV. (**b**) Relationship of the OCV to the absolute differences in AN between the MS and TS. The concentrations of ferro-/ferricyanide salts and tetraethylammonium bromide in the anolytes were 2.5 mM and 50 mM, respectively, whereas those in the catholytes were lower due to the addition of the TS. (**c**) Electrolyte schematic describing the mechanisms explaining the observed increase in the OCV.

A difference in the solvation state of the redox couple between H-TS and L-TS is essential to achieve a large OCV for SDC. Hence, electrochemical reaction systems (a combination of a redox couple, MS, TS or other solutes) mainly affect the OCV. Although there should be many kinds of approaches for obtaining large OCVs, we show a strategy focused on the electron acceptability of solvents, as an example^35^. The ferrocyanide and ferricyanide ions were applied to the redox couple. Previous studies have shown that the half-wave potential of the ferro-/ferricyanide redox couple is largely dependent on the acceptor number (AN) of the solvent^35–38^. Since ferrocyanide ions have a larger negative charge than ferricyanide ions, ferrocyanide ions interact more strongly with solvents with high electron-accepting ability. This results in a positive shift of the redox potential in solvents with a strong electron-accepting ability. Therefore, to obtain a large OCV for a SDC with ferro-/ferricyanide ions, generating a large difference in electron-accepting ability between H-TS and L-TS is expected to be an effective approach. To demonstrate the above strategy, we investigated the OCV of cells by applying four kinds of MS (Fig. 4a). Water was applied as a TS to all four cells, whereas nonaqueous solvents with different electron-accepting abilities (ethanol (EtOH), isopropyl alcohol (IPA), dimethyl sulfoxide (DMSO) and *N*,*N*-dimethyl formamide (DMF)) were applied as the MS. To enhance the solubility of ferro-/ferricyanide salts in nonaqueous solvents, we prepared tetraethylammonium salts of ferro-/ferricyanide^39^. In addition, tetraethylammonium bromide was used as the supporting electrolyte. We investigated the changes in the OCV observed following the addition of TS to H-TS (cathodic side), while the molar ratio of TS at L-TS (anodic side) was fixed at 0.1. The H-shaped cell in Fig. S1 was used for the measurement of the OCV.

Figure 4b shows the relationship between the OCVs and the absolute differences in AN between the MS and TS. For all four cells, an increase in the TS on the cathodic side results in an increase in the OCV. Moreover, a larger OCV is observed in the cells containing DMSO or DMF as the MS. The largest OCV of 0.63 V is observed when DMSO was applied as the MS, and the molar ratio of the TS on the cathodic side is 0.8. Owing to the stronger electron-accepting ability of the TS than that of the MS (Fig. 4a), the addition of the TS to the cathodic side results in positive shifts of the redox potentials of ferro-/ferricyanide ions and an increased OCV (Fig. 4c). In particular, the large OCV in the cells containing DMSO or DMF as the MS is mainly attributed to the large gaps in the electron-accepting ability between H-TS and L-TS, which is caused by the combination of the MS and TS with largely different ANs. These results are in good agreement with our strategy mentioned above. Additionally, we also carried out constant-current discharging tests for the flow-type cell applying DMSO or EtOH as the MS (Fig. S5). For a cell utilizing DMSO as the MS, a large cell voltage is observed during the discharging process, in agreement with the result shown in Fig. 4b. To increase the power output, the structures or materials of cell components should be studied, as previously investigated for redox flow batteries^40–45^.

## Discussion

Although SDCs based on a ferro-/ferricyanide redox couple are shown as an example in this work, the concept of a SDC can be expanded to other electrochemical reaction systems for further improvements and applications. With regard to the OCV, for instance, approaches focusing on the electron-donating property of solvents can be expected^46^. Previous studies have shown a correlation between the electron-donating property of solvents and the redox potentials or free energy of reaction for some redox couples with a positive charge^33,47^. As another example, a previous study has shown that not only solvents but also cations affect the redox potentials of ferro-/ferricyanide ions, implying the possibility of a further increase in the OCV by appropriately selecting counterions, supporting electrolytes or other solutes^36^. These previous studies support the notion that a diversity of strategies can be used to obtain large OCVs. For energy density, the reaction systems should be explored, focusing on other properties in addition to OCVs, including reaction kinetics, solubility of the redox couple, and ion conductivity of the electrolytes. Furthermore, an extension to other reaction systems may provide multiple options for charging processes (Fig. S6). In the above demonstration for the solvation recovery, the phase change process was applied by designing a combination of the MS and TS focusing on the difference in the boiling points. Because the processes should be optional thermodynamically, as mentioned above, other separation and transfer processes can be utilized as charging methods, which can be achieved by designing the reaction systems from another point of view. For instance, different viewpoints from the phase change processes, such as the difference in molecule size between the MS and TS, may be required for applying the charging method based on membrane separation processes^48,49^. Moreover, applications of mechanical processes such as membrane separation may enable the utilization of mechanical energy as an input for charging, which can contribute to the direct use of renewable mechanical energy. Thus, the reaction systems can be designed flexibly for applying various charging processes, which implies new approaches for the utilization of diverse energy sources as an input for the charging of electrochemical cells.

The characteristics of conventional cells, concentration cells, and SDCs are summarized in Table 1. Conventional cells and concentration cells are driven by the difference in standard potentials between two different redox couples and the difference in concentrations, respectively. SDCs are driven by the difference in solvation states of the same redox couple between the anodic and cathodic sides. For the energy input for charging, conventional cells are charged by electrical energy via reversing the reactions that occur during discharging. In SDCs, diverse forms of energy can be used via separation and transfer of the TS. Additionally, electrical energy can also be used for charging by using the same charging method as used in conventional cells. Concentration cells can also be charged by diverse forms of energy because the transfer of solvents can be used to regenerate the difference in the concentrations. However, a large volume of solvent must be transferred to obtain cell voltages that are equivalent those obtained for SDCs. For instance, an open-circuit voltage of 0.172 V was achieved in a previous study by using two aqueous solutions of NaCl at a concentration of 0.017 and 0.513 M, respectively^24^. To regenerate the difference in concentrations via the transfer of solvent after discharging, a large volume of solvent is required to be transferred compared with that in SDCs (Fig. S7). As shown in Fig. 4b, an open-circuit voltage of 0.176 V is obtained in the SDC by applying DMSO as the MS and water as the TS, in which the molar ratio of the TS in the solvent on the H-TS side is 0.3 (the volume ratio is only 0.1). This result indicates that in SDCs, the volume of solvent to be transferred for charging is very small compared with that in concentration cells.

**Table 1:** Comparison of the characteristics of conventional cells, concentration cells, and SDCs.

Electrochemical cell	Driving force for discharge	Charging process	Forms of energy input for charge
Conventional cell	Difference in standard potentials between two different redox couples	Reverse of discharge	Electrical energy
Concentration cell	Difference in concentrations of redox species or electrolytes	Reverse of discharge	Electrical energy
		Transfer of solvent	Thermal energy Mechanical energy etc.
Solvation difference cell (This work)	Difference in solvation states	Reverse of discharge	Electrical energy
		Separation and transfer of TS	Thermal energy Mechanical energy etc.

## Conclusion

In this work, we show the concept of a SDC and an example of electrochemical reaction systems utilizing a ferro-/ferricyanide redox couple. Stable discharging and charging via the newly proposed charging method called solvation recovery is successfully demonstrated in a simple aqueous system. Furthermore, we discuss the dominating factors for the cell voltages for designing high-performance SDCs. The cell voltages are successfully tuned, and a large cell voltage of 0.63 V is achieved, which is consistent with a strategy focusing on the relationship between the redox potential of ferro-/ferricyanide ions and the electron-accepting ability of solvents. The extension of the concept to various electrochemical reaction systems implies the possibility of further improvements in system performance and applications of various charging processes that can allow the utilization of diverse forms of energy as an input for charge, whereas electrical energy must be used for charging of conventional cells. Although concentration cells can also utilize diverse forms of energy for charging via transfer of solvent, the volume of solvent to be transferred to regenerate the required difference in concentrations is estimated to be large compared to a SDC with an equivalent cell voltage. Further developments of electrochemical reaction systems for SDCs can provide new pathways for the use of unutilized energy sources.

## Methods

### Measurement of the OCV

All OCV measurements were conducted using a potentiostat/galvanostat system (SP-50, BioLogic). At the beginning of the measurements, 40 ml of the same L-TS electrolyte was placed into both sections of the H-shaped cell (Fig. S1). The two sections were separated with a 4 mm-thick glass filter. Glassy carbon disc electrodes were inserted into the electrolytes. The values of the cell voltages were recorded every second, and the averages of the values measured for 30 s were plotted. After the measurement, TS was added to one side to increase the molar ratio of TS. The side to which TS was added is defined as the H-TS side. Then, after the molar ratio of the TS on the H-TS side was increased, the OCV was measured. The above procedure was repeated until all the data plotted in Figs. 2b, 4b and S2 were obtained for each cell.

### Discharging and charging via the solvation recovery process

Carbon paper (SIGRACET 38AA, SGL Group) was employed as the electrode for the flow-type cell.

A cation exchange membrane (SELEMION™ CMVN, AGC Group) was employed as the separator of the cell. The electrodes were sealed with PTFE gaskets. Serpentine flow channels for the electrolytes were carved onto graphite plates. Copper plates coated with gold were employed as the current collectors. These components were sandwiched between the two end plates. The volume of the catholyte was 20 ml, whereas that of the anolyte was larger due to the addition of the TS before the first discharge. The electrolytes were supplied to the cell by peristaltic pumps through silicon tubes during discharging. The flow rate of the electrolytes was approximately 13 ml min^−1^ on each side. The discharging current was controlled with the same potentiostat/galvanostat system mentioned above for the OCV measurements. The discharging was continued until the cell voltages were lower than 10 mV.

After the first discharge, the discharged electrolytes were collected from the cell. The two bottles containing the discharged anolyte or catholyte were connected via a PFA tube and silicon tube (Fig. S4). During the solvent recovery process, the discharged anolyte was heated in a hot water bath at a temperature between 69 and 75 °C to evaporate the acetone, whereas the discharged catholyte was cooled in a water bath at a temperature between 20 and 21 °C to recondense the evaporated acetone. The evaporated acetone was transferred to the discharged catholyte through the PFA tube. After trapping the acetone in the discharged catholyte, the gas phase was recirculated to the bottle with the discharged anolyte through the silicon tube by a peristaltic pump to promote the transfer of acetone. After the transfer, the heated anolyte was cooled to room temperature before the second discharging process. For safety, the experimental setup is a partially open system, and, thereby, leads to some loss of acetone during the transfer. However, this loss can be eliminated by designing a closed system with appropriate safety arrangements in practical systems.

## Supplementary Information

Supplementary Information.

## Abbreviations

SDC: Solvation difference cell
MS: Main solvent
TS: Transferred solvent
H-TS: Solvent with a higher TS ratio
L-TS: Solvent with a lower TS ratio
R: Reductant
O: Oxidant
OCV: Open circuit voltage
AN: Acceptor number
EtOH: Ethanol
IPA: Isopropyl alcohol
DMSO: Dimethyl sulfoxide
DMF: *N*,*N*-Dimethyl formamide
