# Supplementary material for: Electrochemical cell recharging by solvent separation and transfer processes
Source: Sci Rep. 2022 Mar 8;12:3739. doi: 10.1038/s41598-022-07573-x (PMC8904837; doi:10.1038/s41598-022-07573-x)
Supplement: Supplementary file 1 — Supplementary Information. [file 41598_2022_7573_MOESM1_ESM.docx]

**Supplementary Information**

**Electrochemical cell recharging by solvent separation and transfer processes**

Yohei Matsui,^1,2,*^ Makoto Kawase,^1^ Takahiro Suzuki,^2^ and Shohji Tsushima^2^

^1^Energy Chemistry Division, Energy Transformation Research Laboratory, Central Research Institute of Electric Power Industry, Yokosuka, 240-0196, Japan.

^2^Department of Mechanical Engineering, Graduate School of Engineering, Osaka University, Suita, 565-0871, Japan.

*y-matsui@criepi.denken.or.jp

**Synthesis of the Electrolytes**

All the reagents used in the following procedures were purchased from Wako Pure Chemical Industries, Ltd.

For the cells utilizing water as the MS and acetone as the TS, ammonium ferro-/ferricyanide aqueous solutions were prepared with the cation exchange process described in a previous study^25^. A cation exchange resin (DOWEX MONOSPHERE 650C (H), Dow Chemical Company) and a 10% ammonia aqueous solution were used for the replacement of potassium ions with ammonium ions in aqueous solutions of potassium ferrocyanide or potassium ferricyanide. The catholytes for the cell containing water as the MS and acetone as the TS were prepared by mixing the ferro-/ferricyanide aqueous solutions after the cation exchange process. The anolytes were prepared by adding acetone to the electrolyte with the same components as the catholyte.

For the cells utilizing nonaqueous solvents as the MS and water as the TS, tetraethylammonium ferro-/ferricyanide aqueous solutions were prepared in the same cation exchange process as the ammonium ferro-/ferricyanide aqueous solutions^25^. Then, tetraethylammonium salts of ferro-/ferricyanide were obtained by heating the aqueous solutions after the cation exchange processes at 70 ℃ in an air atmosphere to remove water to some extent, followed by drying in a vacuum oven. The anolytes for the cell containing DMF, DMSO, IPA or EtOH as the MS and water as the TS were prepared by dissolving the salts and tetraethylammonium bromide in a mixture of the MS and the TS. The catholytes were prepared by adding water to an electrolyte with the same components as the anolyte.

**Figure S1.** Cell schematic for the OCV measurements.

**Figure S2.** Effects of the concentration of the ferro-/ferricyanide redox couple on the change in the redox potentials induced by the addition of acetone. (**a**) Experimental setup for the demonstration. (**b**) Change in the redox potentials of the ferro-/ferricyanide ions observed following the addition of acetone. The concentrations on the right side of the cell are higher than those on the left side of the cell due to the addition of acetone.

**Figure S3.** Cell schematic used for the discharging tests utilizing water as the MS and acetone as the TS.

**Figure S4.** Experimental setup for the demonstration of solvation recovery.

**Figure S5.** Discharging tests for the cell with large cell voltages. (**a**) Cell schematic used for the discharging tests. The cell applies EtOH or DMSO as the MS and water as the TS. An anion exchange membrane (SELEMION™ DSVN, AGC Group) was employed as the separator. (**b**) The OCV and discharging behaviours at a constant current density of 10 A m^-2^ for the flow-type cells. The concentrations of ferro-/ferricyanide ions (tetraethylammonium salts) and tetraethylammonium bromide in the anolytes were 10 mM and 500 mM, respectively, whereas those in the catholytes are lower due to the addition of the TS. The volume of the anolyte was 40 ml, whereas that for the catholyte was larger due to the addition of the TS.

**Figure S6.** Examples of optional solvation recovery processes using thermal energy or mechanical energy.

**Figure S7.** Example of the charging process for a concentration cell via the transfer of water. Assuming the charging and discharging cycles for the concentration cell reported in a previous study^24^ via the transfer of water between the two electrolytes, 4.5 L of water is required to be transferred per 1 L of the high-concentration electrolyte to regenerate the difference in the concentrations of the electrolytes.
